# Single-molecule pull-out manipulation of the shaft of the rotary motor F_1_-ATPase

**DOI:** 10.1038/s41598-019-43903-2

**Published:** 2019-05-15

**Authors:** Tatsuya M. Naito, Tomoko Masaike, Daisuke Nakane, Mitsuhiro Sugawa, Kaoru A. Okada, Takayuki Nishizaka

**Affiliations:** 1 Department of Physics, Faculty of Science, Gakushuin University, Tokyo, 171-8588 Japan; 2 Department of Applied Biological Science, Faculty of Science and Technology, Tokyo University of Science, 2641 Yamazaki, Noda City, Chiba, 278-8510 Japan; 3 Graduate School of Arts & Sciences, The University of Tokyo, 3-8-1 Komaba, Meguro-ku, Tokyo, 153-8902 Japan

**Keywords:** Intracellular movement, Single-molecule biophysics

## Abstract

F_1_-ATPase is a rotary motor protein in which the central γ-subunit rotates inside the cylinder made of α_3_β_3_ subunits. To investigate interactions between the γ shaft and the cylinder at the molecular scale, load was imposed on γ through a polystyrene bead by three-dimensional optical trapping in the direction along which the shaft penetrates the cylinder. Pull-out event was observed under high-load, and thus load-dependency of lifetime of the interaction was estimated. Notably, accumulated counts of lifetime were comprised of fast and slow components. Both components exponentially dropped with imposed loads, suggesting that the binding energy is compensated by the work done by optical trapping. Because the mutant, in which the half of the shaft was deleted, showed only one fast component in the bond lifetime, the slow component is likely due to the native interaction mode held by multiple interfaces.

## Introduction

To dissect how a protein operates as an independent molecular device, the quaternary structure is crucial to realize its unique function. While one structural subunit exhibiting an inherent tertiary structure is essentially responsible for the enzymatic activity, the composition of multiple subunits often orchestrates more complex and high-ordered characters in a single protein. One good example is a molecular motor, myosin: the single ‘subfragment-one’ domain is enough to drive the motility^1^ and the unitary step with ~5 nanometer (nm)^2^, but the function is further enhanced to move without dissociation with larger steps of 36 nm by taking a dimer form as a more complex quaternary structure^3,4^. Another extreme is a rotary molecular motor: engines that are symmetrically arranged induce one-directional rotation of the other component^5^. Thus rotational motion is realized by the cylindrical quaternary structure, like the case of F_1_-ATPase^6,7^ and bacterial flagellar motor^8,9^. Recently, archaellar motor was accepted as the device that works as a rotary motor with a consumption of the chemical energy of ATP hydrolysis^10^ and its characteristics were revealed by biophysical approaches developed for single-molecule observations^11^.

To gain insights into how a quaternary structure of protein that assembles subunits enhances or realizes additional new functions, we here propose a new approach, the direct dismantlement of the subunit composition. Stochastic dissociation of the assembled subunits in aqueous solution occurs in equilibrium, but techniques enabling a forcible disassembly of components possibly reveals the interaction between subunits in a single protein. We took F_1_-ATPase as an example and attempted to impose an external force to pull out the shaft from the cylinder at the single molecular level.

F_1_-ATPase is the part of the F_o_F_1_-ATP synthase, which catalyzes the synthesis of ATP from ADP and inorganic phosphate using proton-motive force across a membrane. The F_1_ sector containing five different subunits, α_3_β_3_γδε, solely hydrolyzes ATP when isolated. Notably, α_3_β_3_γ subcomplex, which we refer hereafter as F_1_-ATPase, is the world’s smallest rotary motor ever found: the γ shaft rotates against the α_3_β_3_ cylinder in a counterclockwise manner when viewed from the protruded side of γ driven by the chemical energy of hydrolysis of ATP. F_1_-ATPase has three catalytic sites at the interfaces between α and β subunits, and thus they are arranged 120° apart around the γ-subunit. It was experimentally proven that the hydrolysis reactions in the three catalytic sites occur not independently but sequentially, one site after another, through simultaneous observation of nucleotide kinetics and rotation under an optical microscope^12^.

In the present study, we pulled the shaft of the single motor through a polystyrene bead by optical trapping^13^ whereas the cylinder was firmly fixed to the glass surface. The bead finally diffused away from the surface after the release of trapping, and thus the lifetime of the linkage was quantified in detail. Although the pull-out of the shaft was not directly visualized in our experimental setup, lifetime measurements of the mutant that lacked half of the shaft indicates the breakage occurred at the interface between the shaft and the cylinder in the single motor. The load-dependent lifetime provide insights about multiple interaction modes of the cylinder with the shaft.

## Results

### Rotation of F_1_-ATPase under low-load and pull-out of shaft from cylinder

**Figure 1:**
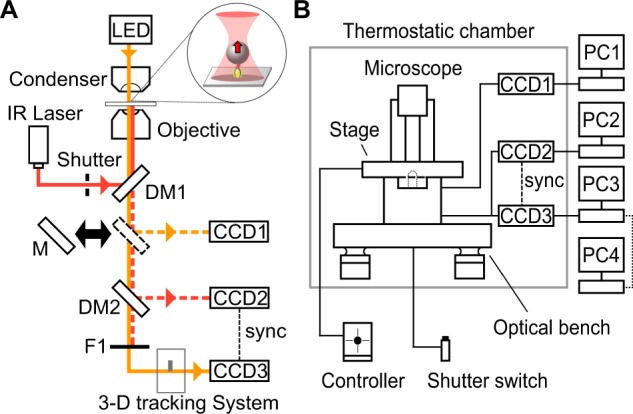
Measurement of 3-D position of the probe under an optical microscope. (**A**) The diagram of optical paths of the experimental system, which includes four components: the optical tweezers (red); the detection of the infrared light (dotted red); the bright-field observation of beads (dotted orange); and the optical system that enabled three-dimensional tracking (orange). (**B**) The schematic of the whole measurement system. The microscope and optical bench were enclosed in the chamber with 20 m^3^ volume that was thermostatically stable with heater and a chiller, and all operations and recordings were done from the outside using four PCs to separate heat sources.

We combined a 3-D measurement system^14,15^ with optical trapping to measure the force along the optical axis of a microscope system (Fig. 1A). The experimental setup allows to impose a constant load to a single protein that is immobilized on the glass surface. To avoid drifting displacement between the trapping position and the sample on the glass, which is mainly caused by the instability of the temperature of the optical system, the whole observation instruments including light sources and the optical bench was enclosed in a circulator-free thermostatic chamber (Fig. 1B). The chamber ensured the temperature to be kept within ±0.2 °C, which allowed only ∼20 nm drifting in *x*-, *y*- and *z*-direction over 10 min. (Fig. S1).

**Figure 2:**
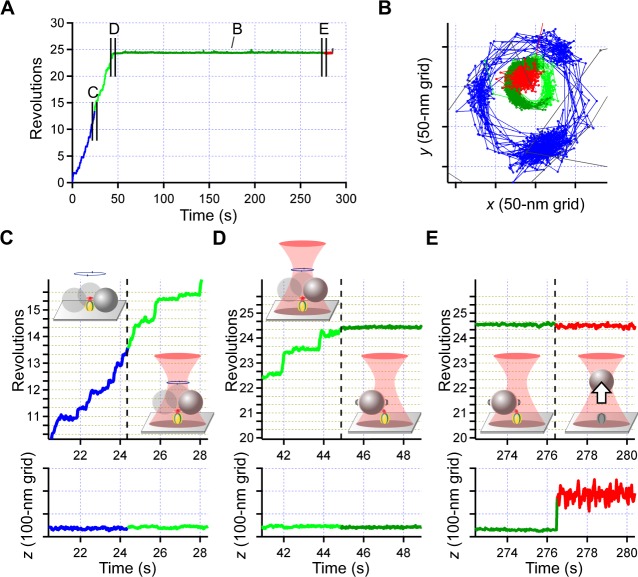
Rotation of F_1_-ATPase under low-load at 100 nM ATP recorded with 33-ms time resolution. (**A**) Time course of rotation. Blue, light green, dark green and red portions correspond to free rotation, rotation under trapping, the pause under trapping and behavior after pulled-out event, respectively. See schematics in (**C**–**E**) for the detail. These color codes are the same with below figures. (**B**) Trace of the bead. (**C**–**E**) Magnified views in *A*.

Under the microscope system described above, we applied an external force to the bead that was anchored to the glass through F_1_-ATPase (Fig. 1A, *inset*). We observed rotation of the shaft of F_1_-ATPase through a single polystyrene bead with the diameter of 0.8 micrometer as a standard rotation assay^12,16,17^. Histidine tags introduced to β subunits were specifically attached to the glass coated with Ni-NTA and so the α_3_β_3_ cylinder was expected to be tightly immobilized onto the glass. The carboxy-modified polystyrene beads were covalently coupled with streptavidin to form avidin-bead. To attach the avidin-bead to the shaft, two residues in the γ subunit were replaced with cysteines as γ-S109C and γ-I212C, and covalently modified with biotins^16,17^. And therefore, the shaft and the surface of the bead were combined through series of covalent bonds and avidin-biotin bonds. When the power of the laser was relatively low, 6–11 mW at the sample plane, and the distance between the bead and the trap center was small, the bead still showed rotation (24.4–44.9 s in Fig. 2A–C). The rotation radius became smaller after trapping (*blue*→*green* in Fig. 2B) but the rotation rate was not altered. The rotation rate before and after trapping was 0.65 ± 0.2 and 0.62 ± 0.2 r.p.s (*n* = 11; average ± s.d.), respectively, suggesting that the condition of our low-load regime did not affect the rotation rate, and thus F_1_-ATPase kept its original function. The decrement of the radius was possibly caused by simple bending of a flexible part that did not directly relate to the sequential catalyses in three sites in the cylinder. Unexpectedly, rotation suddenly stopped in a stochastic manner (see Fig. S3; e.g., 44.9 s in Fig. 2D). We continued the observation and occasionally detected sudden displacement of the bead toward *z*-direction (276.4 s in Fig. 2E). This displacement was the complete dissociation of the bead from the surface, which was clearly identified as the bead diffused away into the aqueous medium after the trapping was released.

We made attempts to pull out the only rotating beads in the presence of 100 nM ATP, which ensured that all samples were anchored to the surface through active F_1_-ATPases. 68% of beads were finally dissociated from the surface within the observation time, 20 min, after trapping in our low-load regime (0.4–2.3 pN; *n* = 41; For remaining 32%, beads did not dissociate and kept linked to the surface, but did not resume rotation). Among them, 34% samples immediately stopped rotation when trapped, and 66% beads stopped after 3–141 s. Therefore, stopping of rotation was required before pull-out event with no exception.

To consider the origin of the release of beads, we explore possibilities of a breakable junction; (i) the attachment between the glass surface and the cylinder (α_3_β_3_ subunit complex) of F_1_-ATPase, or alternatively, (ii) the interaction between the cylinder and the shaft (the γ subunit) in F_1_-ATPase. The first one is not plausible because three large histidine tags were introduced to the three β subunits and the cylinder can specifically take a firm structure with Ni-NTA on the glass surface. We here claim that the sudden displacement of the bead is attributable to the pull-out event of the shaft of F_1_-ATPase from the cylinder (schematic in Fig. 2E). This expectation coincides with the stopping of rotation before pull-out events, as the load directly couples to the interaction between the shaft and cylinder, and finally causes the subsequent dissociation. This scenario is also supported by results obtained by the experiments with a mutant.

### Procedures for lifetime measurement

**Figure 3:**
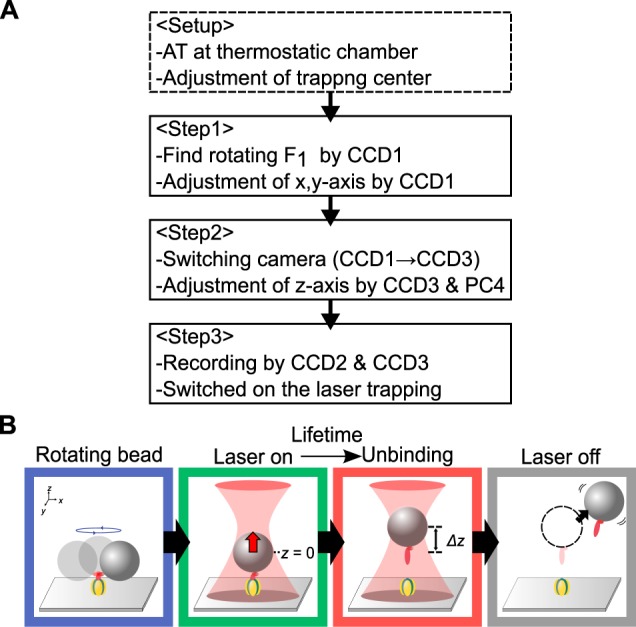
Diagram for the adjustment of the sample stage to measure the lifetime of the bond between the shaft and cylinder. Symbols of CCD and PC are numbered as in Fig. 1A,B. CCD2 and CCD3 are for high-speed recording of infrared light and 3-D, respectively, while CCD1 is for xy-scanning a large area to find rotating beads. PC4 is real-time tracking system for multiple beads with video-rate of NTSC signal, and the display of PC3 that is converted to NTSC is re-captured by PC4 to adjust z-axis of the sample plane. For more detail, see the manuscript. (**B**) Series of schematics showing the measurement procedure. The constant force is applied to the rotating bead anchoring to the glass through single F_1_-ATPase (blue→green). The shaft is subsequently pulled out from the cylinder as a sudden displacement of the bead (green→red). The bead diffused away after the release of the trapping (grey). The lifetime is determined by the period between green and red, and the imposed load is estimated from the Δ*z* in red and the spring constant of optical trapping.

Next, we attempted to measure load-dependency of the lifetime of the interaction between above subunits. In optical trapping, quantification of the applied force to a biomolecule is determined from the distance between the trap center and position of the trapped bead^18^, which is typically within ∼200 nm for *xy* and ∼350 nm for *z* (Fig. S2). The drifting motion of either the trap center or the sample stage thus hinders precise measurement, and so a reproducible manipulation under mechanically stable condition were crucial. We followed detailed procedures as shown in the diagram in Fig. 3A in the condition of our high-load regime. Auto tuning (AT) of the thermostatic chamber was done in advance to determine feedback parameters for the PID regulation. All power supplies of instruments were turned on the day before the experiment to equilibrate the temperature of the chamber. The trapping center was set the position ∼200 nm above the focal plane of the visible light of the objective by the adjustment of the focusing lens of optical-trapping system. For data acquisition, following three steps are required (see Step 1–3 in Fig. 3A diagram). First, rotating probe is identified by CCD1 as 2-D image and *xy*-position of the sample is adjusted to the center of the laser (cf. Fig. 3B *blue*). Second, to manipulate *z*-position of the probe, the image of the probe is guided to the optical system for 3-D tracking system (orange line in Fig. 1A). The tentative display of the high-speed camera CCD3 is re-captured by PC4 equipped with the image-capturing PCI board. By monitoring *z*-position of the bead in real time with an analysis software in PC4, the bead is precisely set to the position ∼200 nm lower than the trapping center. Finally, synchronous recordings of CCD2 for the laser detection and CCD3 of 3-D tracking start, and optical trapping is subsequently applied to the sample (cf. Fig. 3B *green*) by opening the shutter that blocks the laser path. The exact moment when the load started to be imposed to specimens was determined by the signal from CCD2 without any delay. With these procedures, two values, the lifetime of the bond between the shaft and the cylinder (the period between *green* and *red* in Fig. 3B), and the imposed force estimated by the bead displacement (Δ*z* in Fig. 3B), are determined. Pulling-out of the shaft is confirmed by the dissociation of the bead into the medium after that the laser was turned off (*grey* in Fig. 3B).

### Lifetime of bond between shaft and cylinder in F_1_-ATPase

**Figure 4:**
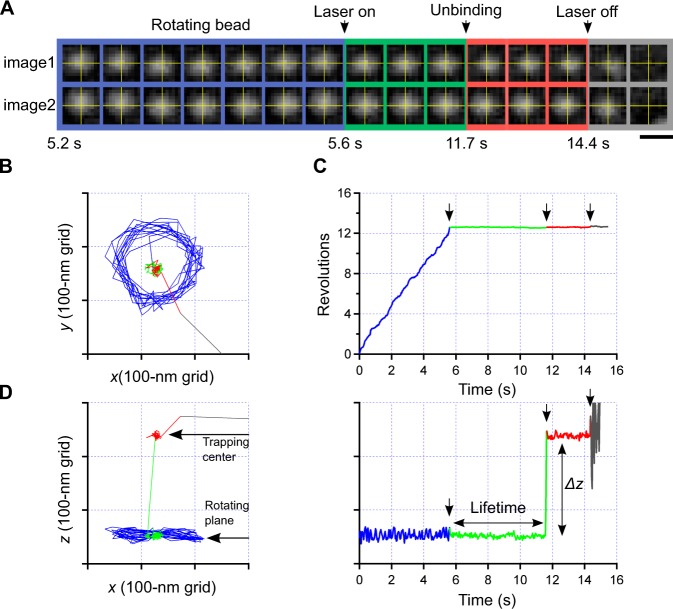
A typical example of unbinding process of the shaft by the load imposed under optical trapping. (**A**) Sequential micrograph of bright field image of the bead attached to the shaft of F_1_-ATPase that is immobilized on the glass surface. Each single image was split into two images (upper and lower) by the prism located at the equivalent back focal plane of the objective. Four color codes, blue, green, red, and grey correspond to periods of rotation without trapping, the pause under trapping, the pause after pull-out of the shaft, and free diffusion without trapping, respectively. The same color codes were used in following (**B**–**D)**, also in schematics in Fig. 3B. Scale bar, 1 μm. (**B**) *X*y-trace of the bead. (**C**) Time course of the bead rotation determined from (**B**). (**D**) *Left*, *Xz*-trace of the bead. *Right*, Time course of the displacement of the bead along *z*-direction. The moment when the shaft was pulled out was clearly identified from this plot.

We measured the lifetime at a high-load regime with different nucleotides: wildtype subcomplex (WT) under 1 μM ATP and 1 mM ATPγS. As the rotation of F_1_ was visualized through 3-D tracking system equipped with a wedge prism to separate a beam flux into two components of light^14,15,19,20^, two images (e.g., Fig. 4A and Movie S1) were always taken on the single camera (CCD3 in Fig. 1A) as raw data. CCW rotation of the bead (*blue*) was recognized by comparing the bead center and yellow crosses that was superimposed for clarity. In our previous paper, it was shown that a rotation radius of the shaft depends on the size of markers^17^. When the bead with the size of 0.8 μm in diameter was applied to the assay, the rotation radius became typically 50–100 nm (cf. blue lines in Fig. 4B). When the bead was trapped (5.6 s), rotation was immediately hindered as the load was high (cf. *green* in Fig. 4A–C). After a short while, the bead was slightly displaced toward *z*-direction (11.7 s). The displacement was recognized even by eye (see relative positions of two images against crosses before and after 11.7 s in Fig. 4A), and precisely quantified with image analyses (Fig. 4D) as Δ*z* = 194 nm in this case. The detachment of the bead from the surface was directly confirmed with the behavior after the release of trapping, i.e., the bead freely diffused away into the medium.

**Figure 5:**
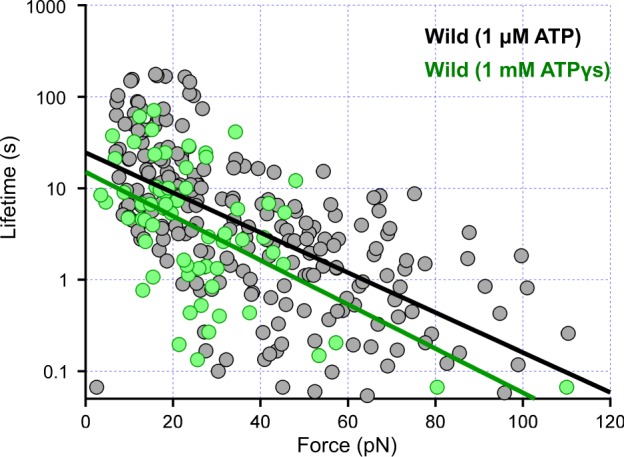
Relationship between the lifetime τ and imposed load *F* in the F_1_-ATPase wildtype under 1 μM ATP (*n* = 219, black) and 1 mM ATPγS (*n* = 64, green). Measurements under low and high-load were recorded with the time resolution of 33 and 2 ms, respectively. Fitting equation is τ(*F*) = τ_0_ × exp(−*F*/*a*), where τ_0_, the lifetime without load, and *a*, the parameter relating the drop of the lifetime against loads. τ_0_ and *a* are 24 s and 20 pN for ATP, and 15 s and 18 pN for ATPγS, respectively.

We performed these pull-out measurements for more than one-hundred samples in the presence of either 1 μM ATP or 1 mM ATPγS. These concentrations were chosen as rotation rate became similar: 2.0 r.p.s. and 2.2 r.p.s. at [ATP] = 1 μM and [ATPγS] = 1 mM, respectively (*n* = 20), which are comparable to previous reports. Note that measured lifetimes should follow an exponential distribution, because unbinding events are stochastic under thermal fluctuation. As expected, raw data were largely distributed as shown in Fig. 5 under both ATP (*n* = 219) and ATPγS (*n* = 64). The exceptional cases (~10%), in which pulling-out event was not detected during recording times (see Methods section in detail), were not included in the plot. To figure out the tendency of the decrement, we fitted an exponential decay, τ(*F*) = τ_0_ × exp(−*F*/*a*), where τ_0_, the lifetime without load, and *a*, the parameter relating the drop of the lifetime against loads, to the experimental data^21,22^. This function did not essentially cover the whole data because several components may be included as shown in the latter analysis. Still, the lifetime roughly dropped an order of magnitude with ~40 pN increment of the load. With this rough estimation, difference of conformational sets of F_1_-ATPase induced by ATP and ATPγS, and thus by inorganic phosphate and monotiophosphate, was not apparent in our measurements (Fig. 5).

### Estimation of lifetimes

**Figure 6:**
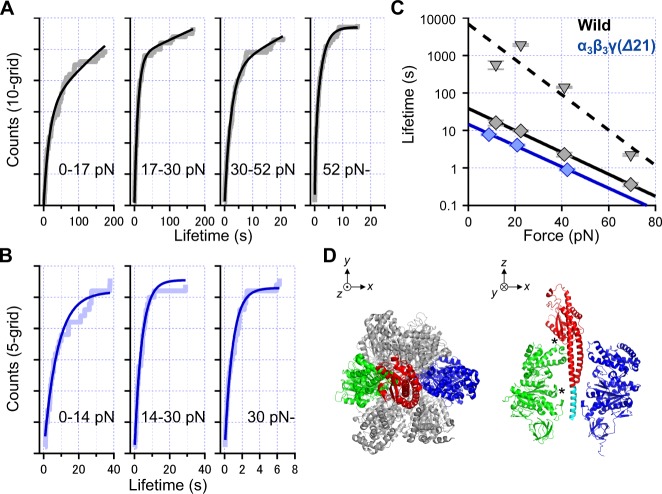
Load-dependency of lifetime of the interaction between the shaft and cylinder. (**A**) The data sets under 1 μM ATP (cf. Fig. 5, black). Four ranges were chosen to distribute the number of data equally; *n* = 50, 57, 54, and 58 for <17, 17–30, 30–52 and >52 pN, respectively. Rectangular lines and curve lines are raw data and fittings as *N* = *N*_sat_ × [1 − *r* × exp(−*t*/τ_s_) − (1 − *r*) × exp(−*t*/τ_f_)] with the slow and fast lifetimes, τ_s_ and τ_f_. In four graphs looking from left to right, the ratio of two populations (*r*) are 0.71, 0.69, 0.65 and 0.69; and the parameter indicating saturated *N* (*N*_sat_) are 105, 154, 125 and 57. (**B**) The accumulated counts of pull-out events of data sets of γ-Δ21 mutant under 1 μM ATP in three ranges of the load; *n* = 28, 27 and 28 for <14, 14–30 and >30 pN. Rectangular lines and curve lines are raw data and fittings *N* = *N*_sat_ × [1 − exp(−*t*/τ)] with a lifetime. *N*_sat_ are 26, 28 and 26. (**C**) Lifetimes estimated from panels in *A* and *B*. τ_s_ and τ_f_ of the wildtype estimated from *A*, and τ of γ-Δ21 estimated from *B*, correspond to black triangle, black diamond, and blue diamond, respectively. Fittings are the function of τ = τ_0_ × exp[−*F* × *d*/(*k*_B_ × *T*)] where τ, the lifetime; τ_0_, the lifetime without any load; *F*, the load; *d*, a parameter having the dimension of length; *k*_B_, Boltzmann constant; and *T*, temperature. Black dashed and solid lines are fitting for slow and fast components, respectively, in the wildtype, while blue solid is for the single component in γ-Δ21 mutant. For error bars, see Materials and Methods section. (**D**) The atomic-structure model of X-ray crystallography 1E79 (ref.^37^). *Left*, top view. *Right*, side view. For clarity, only three subunits, α_E_ (blue), β_TP_ (green) and γ (red and cyan), are displayed. The cyan region of γ are deleted in γ-Δ21 mutant.

To gain more insights into the load-dependency of the lifetime, we divided the load (*x*-axis in Fig. 5) into four ranges of the data set of the sample under 1 μM ATP (*n* = 219) and plotted their accumulated counts to evaluate the lifetime in each range (Fig. 6A). We found that the fitting with a single exponential fitting, *N* = *N*_sat_ × [1 − exp(−*t*/τ)] (cf. Fig. S5), was not appropriate to fit, and thus applied a function with two components of exponentials, *N* = *N*_sat_ × [1 − *r* × exp(−*t*/τ_s_) − (1 − *r*) × exp(−*t*/τ_f_)] where *r* is the ratio of two populations having two different lifetimes, τ_s_ and τ_f_. Both slow and fast lifetimes roughly decreased with the load (black triangle and diamonds in Fig. 6C), suggesting that these analyses properly describe the subunit interaction as a stochastic unbinding event. The implication is that the work done by optical trapping effectively drops the activation energy required to break the interaction. Although we cannot decisively conclude the origin of two populations, τ_s_ and τ_f_ are fitted with the function τ = τ_0_ × exp[−*F* × *d*/(*k*_B_ × *T*)] where τ, the lifetime; τ_0_, the lifetime without any load; *F*, the load; *d*, a parameter having the dimension of length assuming the exponential relationship between the lifetime τ and *F*^21,22^; *k*_B_, Boltzmann constant; and *T*, temperature (black dotted and solid lines in Fig. 6C). τ_0_ and *d* were estimated to be 39 s and 2.8 Å for the fast component, and 6.8 × 10^3^ s and 4.4 Å for the slow component, respectively. The same analysis procedure was also done for the dataset under 1 mM ATPγS (Fig. S4), and confirmed that two lifetimes were similar to those of the dataset under 1 μM ATP.

To address the origin of two components, we attempted to measure the lifetime focusing on the mutant that lacked the bottom half of the shaft, in which twenty-one residues at the C-terminus were deleted, termed as γ-Δ21^23,24^, expecting that two lifetimes might correlate to the different mode of the interaction between the shaft and the cylinder. As previously reported^23,24^, this mutant having the short shaft also exhibits uni-directional rotation. We successfully observed rotation using 792-nm polystyrene bead with 0.90 r.p.s. (*n* = 10) under [ATP] = 1 μM, which are comparable to the above references. Notably, the mutant showed faster lifetimes as compared with the wildtype, and events were plotted as accumulated counts in three ranges (Fig. 6B). Plots could be substantially fitted by the function with a single exponential, *N* = *N*_sat_ × [1 − exp(−*t*/τ)]. Time-constants as τ under different loads were then compared with those of the wildtype (Fig. 6C, blue and black markers). The tendency of the lifetime of γ-Δ21, τ_0_ = 15 s and *d* = 2.7 Å, was similar to that of the fast component of the wildtype, i.e., both τ_0_ and *d* were the same order of magnitude. We also fitted all datasets of both wildtype and γ-Δ21 with alternative fitting (cyan curves in Fig. S5), and concluded that the function involving exponential with single component is enough to fit the lifetime of γ-Δ21, while double exponentials are needed in the case of the wildtype.

## Discussion

Optical trapping is now widely applied to various subjects in biomolecules to measure forces with ranges of the order of 1–100 pN. One limitation in a conventional system equipped with an optical microscope was that forces only parallel to the sample plane are quantified, because the displacement along the optical axis, i.e., the direction of the depth of the objective lens, is hardly measured with the precision of nanometer scale. Our 3-D tracking system successfully overcame the limitation^14,15^, and therefore, the force required to break the bond(s) between the shaft and the cylinder in a molecular rotary motor was measured, for the first time to our knowledge, in the present study. This 3-D measurement system can possibly become the substitute of atomic force microscope (AFM), which is entirely designed to impose the load perpendicular to the sample stage.

We also took the advantage that the force measurement could be done during direct visualization of the activity of enzymes under an optical microscope. We could focus on active and single F_1_-ATPase molecules, because their activity was firmly ensured by the rotation of the marker that was used not only for the detection of rotational motions but also manipulation of molecules. Although the pulling out of the shaft from the cylinder was not directly confirmed in our measurement process, the change in the lifetime of the mutant that lacks half of the shaft strongly indicates that the breakage occurred at the interface between the γ-subunit and α_3_β_3_ cylinder in F_1_-ATPase. In addition, the rotational stop for a significant period of time without exception is consistent with the assumption that a specific intermediate state that leads to breakage between γ and the cylinder is present. To our knowledge, the present study is the first report in which a composition of multiple different subunits is dismantled, not stochastically or chemically, but forcibly by an external force.

In results under the low-load regime exemplified in Fig. 2, rotation stopped before the pull-out event. This observation demonstrated that manipulation of the shaft into upward *z* direction can regulate rotational catalysis through alteration of interactions between the shaft and the catalytic core, such as the case of the manipulation in rotational direction of *xy* by magnetic tweezers^25–27^. The specific state when unbinding occurs is presumably the conformation in which the interactions of γ with β in a surrounding cylinder becomes deficient so as to stop continuous unidirectional rotation of the γ subunit based on the binding change mechanism^28,29^. The mechanism involves correct changes in the nucleotide-binding states that are supported by appropriate conformational changes of the catalytic subunit β^30,31^. Note that there were reports showing long pauses of F_1_-ATPase rotation to regulate their activity without denaturation, such as redox switch inserted in the shaft^32^, the extended form of the ε subunit inserted into the cylinder^33^, mitochondrial IF_1_ protein that interacts with the catalytic β subunit^34^, and ADP inhibition caused by tightly bound ADP at the catalytic site of β^35^. Any similar deviation from authentic conformational pathway of the rotary catalysis based on binding change can lead to a stop, and the stop observed under low-load regime may similarly accompany altered interactions of the catalytic subunit β with the central shaft γ, making the enzyme fall in a state that is not in the catalytic pathway.

Our next focus is specific contact points between β and γ which are necessary for rotation. The deformation would change the number of contact points at the interface and trigger another interaction mode apart from the native state. This scenario coincides with results in Fig. 6, in which the shorter shaft exhibited only one of two components in the lifetime. X-ray crystallography model^6^ showed that there are two contact regions between the shaft and the cylinder (* in Fig. 6D): top C-terminal region of the β-subunit named DELSEED loop with the short helix of γ, and the bottom ‘catch’ named switch II loop in the nucleotide-binding domain of the β-subunit with the C-terminal part of the γ-subunit. In measurements of the wildtype, the deformation might immediately collapse the latter one in ~30% of the samples, and so the component of the fast lifetime might be measured (black diamonds in Fig. 6C) in addition to the slow component (black triangle). In γ-Δ21 mutant, the interaction at the bottom catch did not exist because of the lack of half of the shaft, and therefore, only the fast lifetime was measured (blue diamonds) that coincides with the above scenario. Previous single-molecule rotational studies revealed that the torque generated by the γ-Δ21 mutant is 50% of that of the wild type, and this mutant sometime makes mistakes in rotational direction^24^. Although these characteristics were not judged to be a severe impairment for mechanical rotation itself at that time, the effects were actually not negligible. Our measurements revealed that γ-Δ21 actually lost one of the major contacts between β and γ, and the loss lead to deterioration of function. Taken together with the previous study on γ-Δ21, interaction of the Switch II loop in β with the C-terminal residues in γ represented by the slow component of the lifetime of the present unbinding assay is responsible for a part of the torque generation and correct binding change.

Future studies should be directed at identifying the chemical states of the cylinder portion upon unbinding. It will enable comparisons of lifetime and force between different intermediate states of F_1_ that correspond to different interactions between β and γ. So far, the present 3D tracking microscopy with optical tweezer is the only tool for direct measurements of interaction between the rotor and cylinder subunits in the lateral direction, so the new insights into torque generation can only be obtained through these studies. For example, the reason of low torque for mutants may be clarified by this method as demonstrated by γ-Δ21 mutant in the present study.

As demonstrated in the present study, quantification of forces with our 3-D method combining with optical trapping can be a powerful tool to envisage multiple interaction modes between biomolecules.

## Materials and Methods

### Optical microscopy and cameras

The rotation of a polystyrene bead attaching to the γ-subunit was visualized under an inverted microscope (Ti-E; Nikon) equipped with 100× objective lens (Apo TIRF N.A. 1.49; Nikon), a condenser unit (LWD0.52; Nikon), LED (pE-100 660 nm; CoolLed), IR laser (YLM-2-1064-LP; IPG), an optical bench (RS2000TM; Newport), and three CCDs (*CCD1* in Fig. 1A, CS8430i; Toshiba Teli, *CCD2*, Luca; Andor, and high-speed *CCD3*, LRH20000B; DigiMo). *CCD2* and *CCD3* were synchronized with TTL signal to identify the moment when optical trapping was turned on with a custom modification in *CCD3*. Dichroic mirrors, *DM1* and *DM2* in Fig. 1A, were custom made (Chroma Technology) and purchased (Asahi Spectra), respectively. A highly-stable customized sample-stage (Chukousha) was adjusted three actuators (SGSP-13ACTR; Sigma Koki). The optical system for 3-D tracking system was described previously^14,36^; the calibration factor to determine *z*-position from Δ*x* was set as 1 in all measurements. Most apparatuses except PCs and displays were compartmented in a custom-made thermostatic chamber (Nihon Freezer), and all operations were done from the outside of the chamber. The Measurements were done at 23 ± 0.2 °C. speed of camera was set as 30 and 500 f.p.s. for low and high-load, respectively.

### Preparation and rotation assay of F_1_-ATPase

The α_3_β_3_γ subcomplex of F_1_-ATPase was derived from thermophilic *Bacillus* PS3 as previously described^16,23,24^. For the rotation assay, we used the α(C193S/W463F)_3_β(His10 at N-terminus)_3_γ(S109C/I212C), which is referred to as the ‘wildtype’ in the manuscript. Rotation assay was previously described^12,16,17,30^ with carboxy-modified polystyrene bead (ϕ = 792 nm, Polyscience). The bead was covalently coupled to streptavidin as described previously^31^ with modifications. Briefly, the polystyrene beads were diluted to 1% in 50 mM MES-KOH pH6.1 and then, reacted with 1 mg/mL 1-ethyl-3-(3-dimethylaminopropyl)carbodiimide and 100 mM N-hydroxysulfosuccinimide for 30 min at room temperature. Beads were washed by centrifugation, and the pellet was dissolved in 50 mM MES-KOH (pH6.1). Then, streptavidin (0.1 mg/ml; Sigma-Aldrich) was added to the bead solution and reacted for over 1 h at room temperature in 20 mM potassium phosphate, pH 7.0. The surface of the bottom glass slide of the flow chamber was coated with Ni-NTA as described below. The bottom glass (24 × 32 mm^2^; Matsunami) was soaked in 5 M KOH O.N., washed and immersed in 0.1% 3-mercaptopropyl trimethoxysilane (Tokyo Chemical Industry) in benzene. After washing, the silanized glass was reacted with 36 mg/ml maleimido-C_3_-NTA (Dojindo) in 20 mM HEPES (pH 7.5), 100 mM KCl, 2 mM MgCl_2_ for 3 h. The glass was washed and subsequently soaked in 10 mM NiSO_4_ for 30 min. The resultant Ni-NTA glass was washed and stored in distilled water. To attach F_1_-ATPase to the surface of the bottom glass, the solution with F_1_ was infused into the flow chamber and incubated for three min. After washing by the buffer, the bead solution was added^12,16,17,30^.

### Lifetime measurements

In low-load regime, the movement of beads was recorded at 30 f.p.s. for 1,200 s at maximum. In high-load regime, two conditions were set for different load: 500 f.p.s. for 30 s at maximum under 400–1,200 mW; 30 f.p.s. for 180 s at maximum under 100–400 mW of the laser power. Pull-out events that occurred within 50 ms did not count as a data set because of the limit of the time resolution.

### Analyses

In Fig. 6A, accumulated histograms were fitted with either *f*_1_(*t*) = *N*_sat_ × [1 − *r* × exp(−*k*_slow_ × *t*)− (1 − *r*) × exp(−*t*/τ_fast_)] or *f*_2_(*t*) = *N*_sat_ × [1 − *r* × exp(−*t*/τ_slow_) − (1 − *r*) × exp(−*t*/τ_fast_)] by applying the above function under fitting command in Igor Pro 7 (WaveMetrics, Inc.), where *N*_sat_, *r*, *k*_slow_, τ_fast_ and τ_slow_ were set as variables. *f*_1_(*t*) and *f*_2_(*t*) were used in cases *k*_slow_ < 0.008 and τ_slow_ ≤ 125, respectively, to estimate errors in Fig. 6C. In Fig. 6B, *f*_3_(*t*) = *N*_sat_ × [1 − exp(−t/τ)] was applied to the fitting where *N*_sat_ and τ were set as variables. Deviations of approximated variables acquired through the above procedure were used to depict error values in Fig. 6C.

## Supplementary information

Supplementary Information

Movie S1
