# Supplementary material for: Single-molecule pull-out manipulation of the shaft of the rotary motor F1-ATPase
Source: Sci Rep. 2019 May 15;9:7451. doi: 10.1038/s41598-019-43903-2 (PMC6520343; doi:10.1038/s41598-019-43903-2)
Supplement: Supplementary file 1 — Supplementary Information [file 41598_2019_43903_MOESM1_ESM.pdf]

Supplementary Information

"Single-molecule pull-out manipulation of the shaft of the rotary motor F_1-ATPase"

Tatsuya M. Naito[a], Tomoko Masaike[b], Daisuke Nakane[a], Mitsuhiro Sugawa[c], Kaoru A. Okada[a] & Takayuki Nishizaka[a]

[a]Department of Physics, Faculty of Science, Gakushuin University, Tokyo, Japan; [b]Department of Applied Biological Science, Faculty of Science and Technology, Tokyo University of Science, 2641 Yamazaki, Chiba 278-8510, Japan; [c]Graduate School of Arts & Sciences, The University of Tokyo, 3-8-1 Komaba, Meguro-ku, Tokyo 153-8902, Japan.

This file includes:

Supplementary Figures S1-S5

Captions for Supplementary Videos S1

Supplementary Figure and Legends

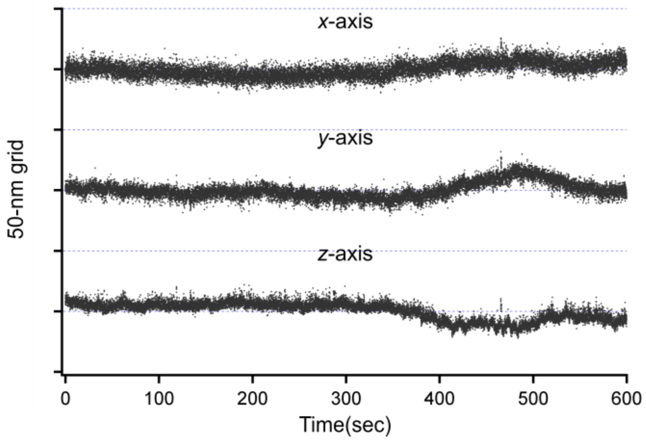

Fig. S1. Stability of the experimental system in a thermostatic chamber. A bead (φ = 800 nm) fixed on a glass surface was tracked under the highly-stable customized sample stage with the time resolution of 30 fps. Upper, middle and lower traces represent the displacements along the *x*, *y* and *z* axes, respectively.

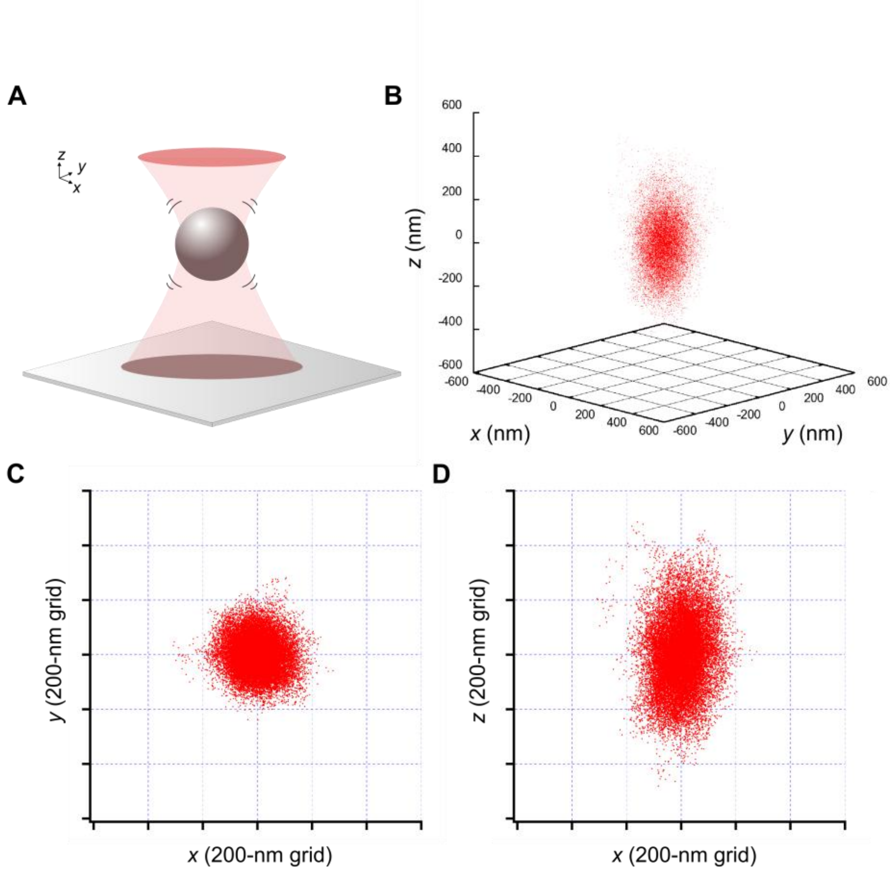

Fig. S2. 3-D Brownian motion of a trapped bead under a low laser-power. (*A*) Schematic. (B) 3-D representation. (C) *Xy*-plot. (D) *Xz*-plot. The trap stiffness along *z*-axis is smaller than that of *x* and *y* as shown by the broader distribution of the bead. Spring constants were estimated as 1.54-1.69 pN nm^{-1} W^{-1} for *x*-axis, 1.55-1.62 pN nm^{-1} W^{-1} for *y* and 0.45-0.49 pN nm^{-1} W^{-1} for z (*n* = 9), where the laser power was measured at the bottom of the objective lens.

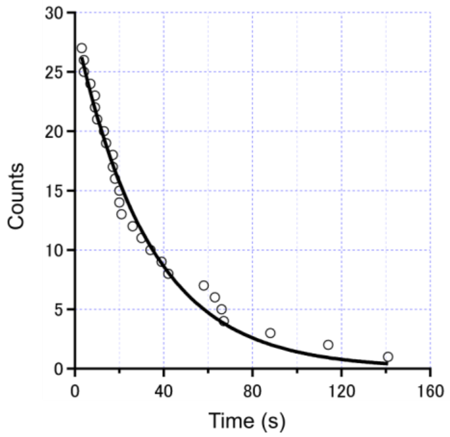

Fig. S3. Lifetime of active state in F_1 under low-load regime (cf. Fig. 2 in the manuscript). The duration time after imposing of the load was plotted as an accumulation histogram (open circle), which was fitted with an exponential function, $N(t) = N_0 \cdot \exp(-t/\tau)$, where N = 29 and τ = 33 s (black line).

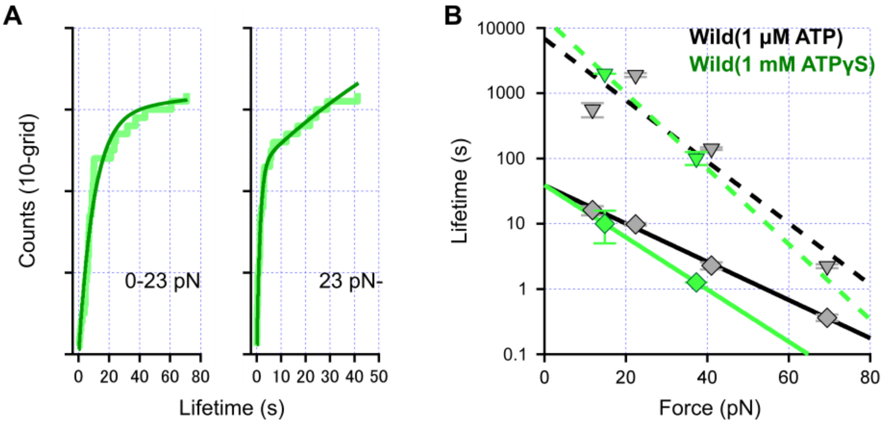

Fig. S4. Load-dependency of lifetime of the interaction between the shaft and cylinder. (*A*) The data sets of wildtype under 1 mM ATPγS (cf. Fig. 5, green) and plotted their accumulated counts to evaluate the lifetime in each range. Two ranges, <23 and >23 pN, were chosen to distribute the number of data equally ($n = 32$). Green rectangular lines are accumulated counts. Fittings (dark green) are $N = N_{sat} \times [1 - r \times \exp(-t/\tau_s) - (1-r) \times \exp(-t/\tau_f)]$, where N_{sat}, parameter to normalize data; r, ratio of two components; τ_s, slow lifetime; τ_f fast lifetime. In *left* and *right* panels, N_{sat} are 75 and 55; r, 0.60 and 0.58, respectively. Estimated τ_s and τ_f are plotted in *B*. (*B*) τ_s and τ_f of wildtype under 1 μM ATP (black) and 1 mM ATPγS (green), latter of which were estimated from *A*. Fittings are the function of $\tau = \tau_0 \times \exp[-F \times d/(k_B \times T)]$ where τ, the lifetime; τ_0, the lifetime without any load; F, the load; d, a parameter having the dimension of length; k_B, Boltzmann constant; and T, temperature. τ_0 and d of the wildtype under 1 mM ATPγS were estimated to be 39 s and 3.8 Å for the fast component, and 1.4×10^4 s and 5.4 Å for the slow component, respectively. Difference of conformational sets of F_1-ATPase induced by ATP and ATPγS, and thus by inorganic phosphate and monotiophosphate, was not apparent in our measurements.

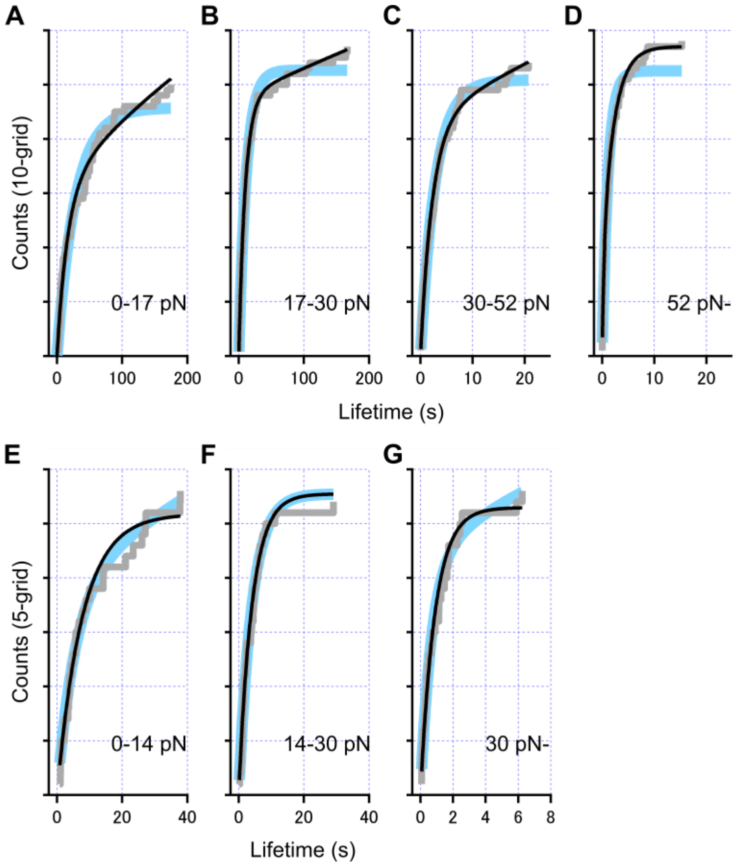

Fig. S5. Accumulated counts of pull-out events of both the wildtype (*A*-*D*) and the γ-Δ21 mutant (*E*-*G*). Rectangle lines (grey) and black lines are the same datasets and fittings, respectively, with those in Fig. 6 *A* and *B* in the main text. Additional fitting curves (cyan) are overlaid to compare with the original representation. (*A*-*D*) $N = N_{sat} \times [1 - \exp(-t/\tau)]$. These cyan fitting curves with single components rose more leftward than black curves with double components, indicating that data points with slow lifetime were not fitted well by the function. (*E*-*G*) $N = N_{sat} \times [1 - r \times \exp(-t/\tau_s) - (1-r) \times \exp(-t/\tau_f)]$. Black curves with single components nearly follow the cyan curves with two components, indicating that the function involving only one lifetime is enough to approximate the dataset in the γ-Δ21 mutant.

Captions for Supplementary Movies

Movie S1. A typical example of pull-out event of the shaft from the cylinder by optical trapping (cf. Fig. 4). Each single image was split into two images (upper and lower) by the prism located at the equivalent back focal plane of the objective. When the bead was trapped (5.6 s), rotation immediately stopped as the load was high. Subsequently, the bead was slightly displaced toward z-direction (11.7 s), which directly indicated the pull-out. The detachment of the bead from the surface was directly confirmed through the behavior after the release of trapping, i.e., the bead was freely diffused away into the medium (14.4 s). Area: 2.3×4.6 μm.
